# Interkingdom Detection of Bacterial Quorum-Sensing Molecules by Mammalian Taste Receptors

**DOI:** 10.3390/microorganisms11051295

**Published:** 2023-05-16

**Authors:** Yobouet Ines Kouakou, Robert J. Lee

**Affiliations:** 1 Department of Otorhinolaryngology, Perelman School of Medicine, University of Pennsylvania, Philadelphia, PA 19104, USA; yobouet.kouakou@pennmedicine.upenn.edu; 2 Department of Otorhinolaryngology and Physiology, Perelman School of Medicine, University of Pennsylvania, Philadelphia, PA 19104, USA

**Keywords:** acyl-homoserine lactone, quinolone, competence-stimulating peptide, *Pseudomonas aeruginosa*, *Staphylococcus aureus*, *Streptococcus mutans*, nasal epithelium, gingival epithelial cells, cilia, solitary chemosensory cell

## Abstract

Bitter and sweet taste G protein-coupled receptors (known as T2Rs and T1Rs, respectively) were originally identified in type II taste cells on the tongue, where they signal perception of bitter and sweet tastes, respectively. Over the past ~15 years, taste receptors have been identified in cells all over the body, demonstrating a more general chemosensory role beyond taste. Bitter and sweet taste receptors regulate gut epithelial function, pancreatic β cell secretion, thyroid hormone secretion, adipocyte function, and many other processes. Emerging data from a variety of tissues suggest that taste receptors are also used by mammalian cells to “eavesdrop” on bacterial communications. These receptors are activated by several quorum-sensing molecules, including acyl-homoserine lactones and quinolones from Gram-negative bacteria such as *Pseudomonas aeruginosa*, competence stimulating peptides from *Streptococcus mutans*, and D-amino acids from *Staphylococcus aureus*. Taste receptors are an arm of immune surveillance similar to Toll-like receptors and other pattern recognition receptors. Because they are activated by quorum-sensing molecules, taste receptors report information about microbial population density based on the chemical composition of the extracellular environment. This review summarizes current knowledge of bacterial activation of taste receptors and identifies important questions remaining in this field.

## 1. Introduction

The immune system has been referred to as our “sixth sense” [1,2]. Like our senses of sight, smell, hearing, touch, and taste, the immune system constantly surveils our environment, with the unique goal of detecting dangerous pathogens. Looking at immunity from this angle, it is not surprising that our immune system uses multiple types of chemosensory G protein-coupled receptors (GPCRs). For example, olfactory (odorant) receptors were originally identified due to their role in detection of smell by olfactory neurons [3] but have more recently been reported to also be expressed in immune cells such as macrophages [4,5]. These olfactory receptors can regulate macrophage polarization or other immune responses [6,7,8,9,10,11].

In fact, human olfactory receptors have been suggested to predate the human sense of smell [12]. In other words, at least some olfactory receptors may have evolved first for non-smell chemosensory roles and were then re-used by the olfactory system. Because hundreds of olfactory receptors exist in the human genome that allow us to discriminate perhaps up to a trillion different odors [12] and only a small fraction have been so far found outside the nose [13], it is likely that we know very little about how this largest class of GPCRs affects human physiology beyond smell. Regardless, it is already established that olfactory receptors are used by the immune system to surveil the microenvironment and likely detect small molecules from invading pathogens and/or the commensal microbiome.

The focus of this review is on the immune roles of another type of sensory receptor: taste GPCRs. Taste receptors were originally identified on the tongue [14] but also more recently have been identified as being expressed all over the body [15,16,17,18]. As described below, the number of taste GPCRs is much smaller than olfactory receptors. Nonetheless, our knowledge of how these receptors function in cells outside the tongue probably remains in its infancy. Similar to olfactory receptors, our immune system uses taste receptors to detect chemical signals from invading pathogens and/or commensal colonizers. Multiple cell types in a variety of different tissues have been shown to use taste receptors to detect several types of bacterial quorum-sensing molecules to regulate a diverse array of immune responses, as described below. The purpose of this review is to summarize our current knowledge of taste receptor–bacterial interactions as well as to highlight important remaining questions in this emerging field.

## 2. Bacterial Quorum Sensing

Quorum sensing is a method of intercellular communication used by individuals of a bacterial species to communicate with each other, with other species, and even to coordinate interactions with their host [19]. It is involved in various bacterial functions in both Gram positive and negative bacteria, such as expression of virulence factors and evasion of immune response [19]. Quorum sensing relies on the production, release, and recognition of signaling molecules, often called auto-inducers [20,21,22,23,24]. When these extracellular quorum-sensing molecules reach a concentration threshold high enough to be recognized by bacterial receptor proteins, which directly reflects the bacterial density, the expression of specific genes is turned on or off. This prevents activation of certain pathogenic responses until the bacterial population is at a high enough density [20,21,22,23,24]. Genes regulated by quorum sensing often include virulence factors such as toxins or proteases that break down host tissue [20,21,22,23,24].

Quorum sensing is notably involved in regulating the formation of complex aggregate communities known as biofilms [20,21,22,23,24]. Aggregated bacteria in biofilms can attach to the surfaces or become embedded in the extracellular matrix [20,21,22,23,24]. Bacterial biofilm formation increases their tolerance to stress and antibiotics [25]. The presence of biofilms enhances the virulence, pathogenicity, and even life-threatening nature of infections, especially in patients who are immunocompromised [26]. Inhibition of quorum sensing (sometimes termed “quorum quenching”) is an actively emerging strategy to prevent biofilm growth in *Pseudomonas aeruginosa* or *Staphylococcus aureus* infections [23,24,27].

Several families of bacterial quorum-sensing molecules have been reported. Gram-negative bacteria such as *P. aeruginosa* and *Escherichia coli* produce and use various acyl-homoserine lactones (AHLs) as their primary quorum-sensing signals. Although they share a common homoserine lactone (HSL) ring, these molecules are structurally diverse. Thus, each AHL is synthetized by a dedicated AHL synthase. *P. aeruginosa*, for example, produces two AHLs, 3-oxo-C12-HSL and C4-HSL, which are synthesized by two independent enzymes, LasI and RhlI, respectively [19]. AHLs specifically interact with “R-proteins” receptors, a class of transcription factors with a DNA-binding domain on their C-terminal end, to stimulate or repress the transcription of various genes [19].

Quorum-sensing signals in Gram-positive bacteria are typically oligopeptides (also called pheromones), such as autoinducing peptides (AIP) [20]. Competence stimulating peptides (CSPs) are notable AIP that are produced and used as quorum-sensing signals by several streptococcal species including *Streptococcus mutans*, *Streptococcus pneumoniae*, *Streptococcus gordonii*, and *Streptococcus intermedius* [28,29]. In *S. mutans*, the CSP-mediated quorum-sensing system is encoded by the *ComCDE* genes and includes the pheromone itself (ComC) and a two-component system consisting of a membrane-bound histidine kinase receptor (ComD) and its cytoplasmic cognate response regulator (ComE) [28,30,31]. ComC is produced as a propetide that must be processed and secreted as a 21-redisue peptide by a dedicated ABC transporter complex (ComAB). The 21-residue peptide is then cleaved at its C-terminal end by a membrane-bound protease, SepM, to form the 18-residue active pheromone. Binding of the active CSP to its receptor ComD results in phosphorylation of ComE and subsequent stimulation of the expression of various genes involved in the acquisition of bacterial genetic competence, biofilm formation, swarming and production of virulence factors [28].

Other notable examples of quorum-sensing signals include the autoinducer-2 (AI-2) found in both Gram-positive and Gram-negative bacteria, the *Pseudomonas* quinolone signal (PQS), and the diffusible signal factor (DSF) [19,26]. The known roles of taste receptors in the host detection of AHL and CSP quorum-sensing molecules produced by invading bacteria is detailed below. However, further research is needed to understand whether taste receptors detect any of the other numerous metabolites and signaling molecules produced by Gram-positive and Gram-negative bacteria.

## 3. GPCR Taste Receptors

Our perception of food is referred to as flavor, which is a combination of smell, mouthfeel (texture), taste, and other stimuli. There are only five canonical tastes detected by the taste bud cells of the tongue: bitter, sweet, savory (also termed “umami”), salty, and sour. The salty and sour tastes are mediated by Na^+^ and H^+^ ion channels, respectively, expressed in distinct subsets of morphologically defined type III cells of the taste bud [32,33]. Salty is detected via the epithelial sodium channel (ENaC) [33]. Sour is detected by H^+^ channel Otop1 [34,35,36]. Activation of either ENaC or Otop1 results in depolarization of the salt- or acid-specific taste cell, which in turn activates the appropriate gustatory neuron to transmit the salty or sour signal to the brain [37].

Instead of ion channels, GPCRs are used to detect bitter, sweet, and umami compounds [38,39]. GPCRs are seven transmembrane domain proteins that change conformation upon ligand binding, setting off intracellular signal cascades [40,41]. Bitter, sweet, and umami GPCRs are expressed in distinct type II taste cells of the taste bud. The GPCR expression, described below, dictates whether the cell is a bitter-, sweet-, or umami-responsive cell. Each type II taste cell detects one type of taste; the coupling of that cell to a specific gustatory neuron codes how the cell response is perceived by the brain [38].

**Figure 1:**
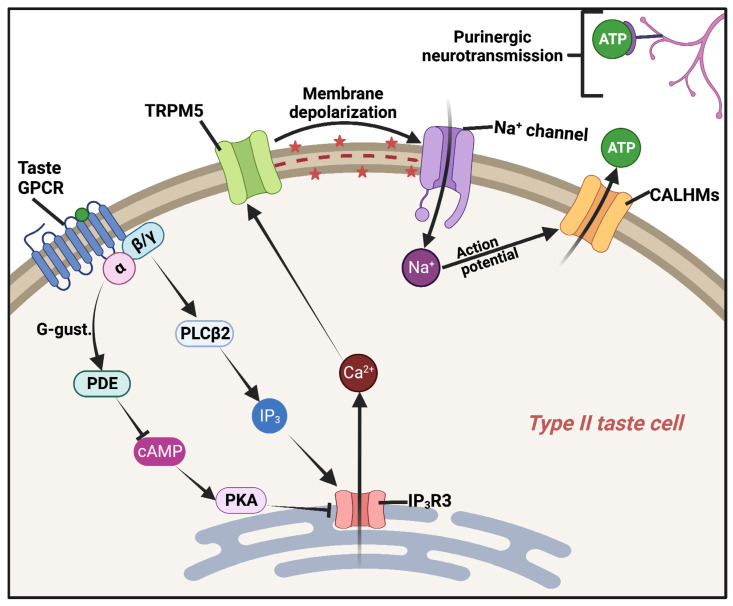
GPCR taste signal transduction pathway in a type II taste cell, as described in the text. Diagram created with BioRender.

Activation of taste GPCRs in the type 2 taste cells likely first results in Ca^2+^ signaling through its G-protein pathways (Figure 1). Gβγ activates the β2 isoform of phospholipase C (PLCβ2) to produce inositol 1,4,5-trisphosphate (IP_3_), which binds to and activates the IP_3_ receptor (IP_3_R). The IP_3_R is an endoplasmic reticulum (ER) ion channel that allows calcium (Ca^2+^) release from intracellular ER Ca^2+^ stores [42,43]. Simultaneously, transducin G protein family member Gα-gustducin activates phosphodiesterase (PDE) activity to reduce cyclic-AMP (cAMP) and decrease activation of protein kinase A (PKA) [44]. It is hypothesized that PKA in taste cells phosphorylates and inhibits type III IP_3_R [45,46], the major IP_3_R isoform found in type II taste cells [47,48,49]. Thus, reducing PKA activity relieves this inhibition to enhance IP_3_R Ca^2+^ release. While this makes sense in the context of taste, it is somewhat surprising as further studies have suggested that PKA enhances IP_3_R activity [50,51,52] or alters IP_3_R release kinetics [46,53] rather than inhibiting IP_3_R activity [45]. In the authors’ opinions, the full role of gustducin activation of PDE in taste signal transduction may not yet be known and may even extend beyond IP_3_R.

Nonetheless, Ca^2+^ release from the ER activates a plasma-membrane-localized cation channel, TRPM5 [54,55]. This causes plasma membrane depolarization which activates voltage-gated sodium (Na^+^) channels [56] to generate action potentials. The end effect is non-vesicular ATP release [57] through a channel complex of CALHM1, CALHM3, and perhaps other CALHM subunits [58,59,60]. This ATP probably originates in large part from large “atypical” mitochondria with large tubular cristae situated close to the plasma membrane in close proximity to the CALHM channels [35,61]. The release of cytosolic ATP through CALHM channels into the extracellular space then activates purinergic receptors on gustatory sensory neurons [62,63].

There are two main families of taste GPCRs. The taste family 1 (known as Tas1R or T1R) group contains three T1R isoforms, T1R1, T1R2, and T1R3 [64,65,66]. T1R receptors are encoded by *TAS1R* genes. T1Rs form sweet and umami receptors, thought to be “pleasant” tastes because they signal the presence of beneficial nutrients in foods [38,67]. The sweet receptor is formed from a heterodimer of T1R2 and T1R3 (T1R2/3), while the umami receptor is created by heterodimerization of T1R1 and T1R3 (T1R1/3) [38,67]. T1Rs are class C GPCRs with N-terminal “Venus fly trap” domains containing binding sites for multiple structurally-diverse agonists [68]. The sweet receptor (T1R2/3) is activated by sugars [69,70,71,72], artificial sweeteners [73], as well as some D-stereoisomer amino acids [72,74]. The umami receptor (T1R1/3) is activated by savory amino acids such as L-glutamate [75]. Umami activation is strongly enhanced by ribonucleotides including inosine monophosphate and guanosine monophosphate [75]. Some type II taste cells have been reported to express only T1R3 without T1R1 or T1R2 [66,76], and T1R3 homodimers may also act as glucose [77,78,79] or Ca^2+^/Mg^2+^ receptors [80], though this remains controversial. A recent study also suggests that Cl^-^ ions at low mM concentrations may bind to T1R3 and evoke some umami and/or sweet taste signaling in mice [81]. Because differences in agonist (aspartame [82]) activation and antagonist (lactisole [83,84], gymnemic acid [85]) inhibition have been reported for mouse and human T1R2/3 sweet receptors, pharmacological experiments carried out in one species may not perfectly translate to the other.

**Figure 2:**
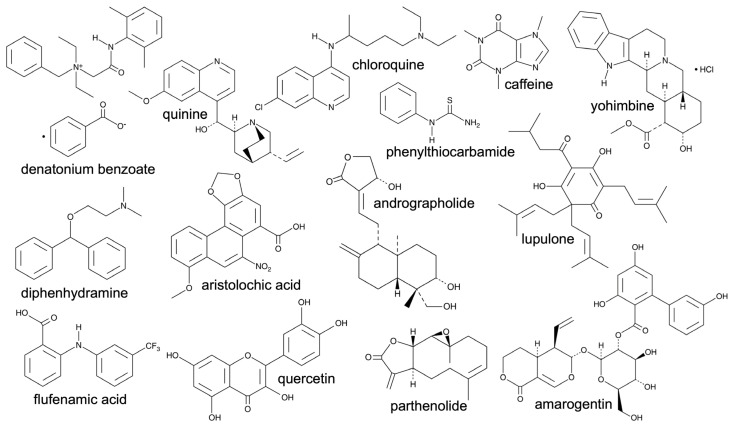
Bitter compounds are structurally diverse. Shown are several structures of representative bitter compounds. An actively-maintained online database of bitter compounds (BitterDB [88]) contains over 1000 structurally diverse compounds shown to activate specific T2R isoforms in various cell models. Some bitter compounds exhibit a high degree of promiscuity among receptors (e.g., denatonium benzoate activates eight human T2Rs and quinine activates eleven T2Rs), while others are recognized by only one T2R (e.g., flufanamic acid activates T2R14 and phenylthiocarbamide (PTC) activates T2R38). An example of the structural diversity of compounds that can activate a single T2R is seen in the structures of diphenhydramine, aristolochic acid, quercetin, parthenolide, chloroquine, and lupulone, all of which can activate T2R14 [88,89]. In addition to small molecules, some proteins and peptides have also been shown to activate T2Rs [88]. This diversity can make predicting the “bitterness” or receptor specificity of specific bacterial metabolites very difficult without empirical testing. Bitter compounds shown were shown to activate the following human T2Rs in heterologous expression models [88,89]: denatonium benzoate, T2Rs4, 8, 10, 13, 39, 43, 46, 47; quinine, T2Rs4, 7, 10, 14, 39, 40, 43, 44, 46; diphenhydramine, T2Rs14, 40; flufenamic acid, T2R14; aristolochic acid, T2Rs14, 43; quercetin, T2R14; chloroquine, T2Rs3, 7, 10, 14, 39; phenylthiocarbamide, T2R38; andrographolide, T2Rs46, 47, 50; parthenolide, T2Rs1, 4, 8, 10, 14, 44, 46; caffeine, T2Rs7, 10, 14, 43, 46; yohimbine, T2Rs1, 4, 10, 38, 46; lupulone, T2Rs1, 14; amarogentin, T2Rs1, 4, 39, 43, 46, 47, 50.

The taste family 2 receptors, Tas2R or T2R, mediate bitter taste and are encoded by *TAS2R* genes [38]. Twenty-six functional T2R isoforms are currently described in humans, with the functional variant of T2R2 only being expressed in some African populations [86]. Much like for the T1R family, T2R isoforms may form heterodimers [87], but the functional consequences of these dimers remain unknown. The diversity of the T2R family allows detection of a wide array of bitter compounds (Figure 2), which could provide protection against consumption of a large variety of toxic bitter molecules found in plants, for instance [38].

The expression, functionality, number, and diversity of taste receptors in different species have been greatly shaped by evolutionary pressure [90,91,92]. These inter-species differences are the result of genetic deletions, duplications, and pseudogenizations. Cats, for example, lost their functional *TAS1R2* receptors during evolution and are unable to taste sweet sugars, likely because their natural carnivorous diet contains very little of these nutrients [90]. Herbivores, on another hand, have an increased repertoire of bitter taste receptors, which allows them to better detect toxic bitter compounds in the plants they consume [92].

At the species level, the expression and function of taste receptors between different individuals is influenced by genetic polymorphisms. Within the T2R bitter taste receptors family, T2R38 is the most well documented isoform. The gene encoding for this isoform, *TAS2R38*, has several single-nucleotide polymorphisms (SNPs) resulting in proteins with different amino acids at positions 49, 262, and 296 [93,94]. Two of these polymorphisms are common in Caucasians. The first one encodes for a functional receptor which contains proline (P), alanine (A), and valine (V) residues at these positions, respectively. The second polymorphism encodes for a nonfunctional receptor that contains alanine (A), valine (V), and isoleucine (I) [93]. For the sake of simplicity, the functional and nonfunctional polymorphisms are referred to as the PAV and AVI variants, respectively. The loss of the valine at position 262 in the AVI variant is probably responsible for the nonfunctionally of the receptor by preventing activation upon binding to its ligands [95,96,97].

In Caucasian populations, the *TAS2R38* polymorphisms resulting in PAV and AVI variants closely follow a Mendelian inheritance pattern. Individuals with the AVI/AVI genotype are “non-tasters” for T2R38-specific agonists such as phenylthiocarbamide (PTC or phenylthiourea PTU) and 6-propyl-2-thiouracil (PROP) [93]. They represent about 30% of this population. In contrast, PAV/PAV homozygous individuals perceive these agonists as extremely bitter upon ingestion and are therefore called “super-tasters”. They make up about 20% of Caucasians. PAV/AVI heterozygous individuals exhibit variable intermediate taste levels [93,98]. Several other variants including the AAI (alanine, alanine, isoleucine) variant (nonfunctional and more common in people of African descent) have been described in other populations [94], but they are extremely rare (<5% frequency) [94]. T2R38 is also involved in the detection of other bitter agonists such as isothiocyanate compounds found in green leafy vegetables. *TAS2R38* polymorphisms may therefore have an impact on how people perceive the taste of these vegetables and on their individual preferences.

The expression of T2R38 receptors outside the oral sphere suggests that *TAS2R38* polymorphisms may have clinical implications beyond taste detection and processing. This observation may also apply to hundreds of other *TAS2R* and *TAS1R* polymorphisms described in humans [99,100] whose phenotypic effects have been only minimally characterized in contrast to *TASR38*. One specific example is the T1R sweet receptor polymorphism involving a valine or isoleucine residue at position 191. Individuals homozygous for the valine variant may be more susceptible to higher absorption of carbohydrate during meals, as well as have an increased risk of hypertriglyceridemia and dental caries [101,102]. The involvement of sweet taste receptors in other biological systems such as innate immunity [103,104], memory and learning-related neuronal functions [105,106,107,108,109], and insulin production by pancreatic beta cells [77,78,110,111,112,113] underscores the need to better characterize *TAS1R* polymorphisms as they may influence these functions beyond diet-related sugar ingestion.

In addition to bitter, sweet, and umami, the tongue may also be able to detect other tastes through GPCRs, including fatty [114,115,116,117,118], metallic [80,119,120], and “kokumi” [121,122,123] tastes. While controversial and harder to characterize, their study is of growing interest in the field of sensory science [124]. Fat is involved in the “texture” component of flavor, and omega-3 fatty acids are known to activate certain GPCRs (GPR40 and GPR120) [114,115,116,117,118]. Some studies have also shown that high concentrations of metal ions can activate T2Rs [125]. In the two previous cases, the consequences of this GPCR activation on the tongue have not yet been elucidated [114,115,126,127,128,129]. Kokumi taste refers to the putative activation of the extracellular Ca^2+^-sensing receptor (CaSR) by various food compounds (peptides, vitamins, minerals) [123]. Kokumi compounds are generally described as tasteless when ingested alone, but when combined with other food, they enhance sweet, salty, and umami tastes. While other GPCR tastes may exist, in this review, we will focus on the interaction between bacteria-produced quorum-sensing signals and the better studied bitter and sweet taste GPCRs [121,122,123].

## 4. “Extraoral” Taste Receptors as Immune Detectors for Quorum-Sensing Molecules

Taste receptors were first described as sensory receptors located on the tongue, where they are expressed in taste cells of taste buds. However, bitter and sweet G-protein coupled taste receptors have recently been identified in other tissues ranging from the lungs and gut to the brain [15,17]. In contrast to what has been described on the tongue, so-called “extraoral” sweet and bitter taste receptors can be co-expressed in chemosensory cell types, such as intestinal tuft cells that regulate anti-parasite immunity [130,131] and solitary chemosensory cells in mouse and human airways [74,104,132,133,134].

The purpose of these seemingly misplaced, extraoral taste receptors was at first baffling, but it is now known that taste is only part of the responsibility of these receptors. Bitter and sweet receptors serve more general chemosensory roles in many tissues, making them potential therapeutic targets or possibly important mediators of off-target drug effects [135], particularly as many medications in clinical use taste bitter [136,137,138]. GPCR taste receptors have been found in a large variety of extra-oral tissues, including but not limited to the airway, brain, lungs, testes, and colon [17]. Extra-oral taste receptor expression may be an early evolutionarily chemosensory development, as *TAS2R* gene expression was detected in both oral cavity (jaws and gills) and other organs (liver and fins) in the teleost cavefish *Astyanax mexicanus* [139]. Fish are the evolutionarily earliest animal containing the *TAS1R* and *TAS2R* genes used by vertebrates for bitter, sweet, and umami tastes [140].

It is important to note that these extra-oral taste receptors do not mediate “taste” per se as they are not linked to neuronal perceptive pathways, but they still serve as local chemoreceptors in the body. The known distribution of bitter and sweet taste receptors varies between organs, it is thought that some express only bitter or only sweet receptors, while others express both. We are only beginning to understand the diverse roles of these receptors. For example, sweet taste receptors in the pancreas and intestine may regulate insulin secretion [78,110,111,141], and glucose transporter expression [142,143,144], respectively, in response to glucose. Bitter taste receptors in the male reproductive system are important for fertility [145,146,147].

As described in detail in the following sections, one component of extraoral taste receptor function is the detection of bacterial quorum-sensing molecules (Table 1). We primarily focus on the oral and nasal epithelium, but taste receptors also play an immune role in the gastrointestinal tract [25,27]. Of particular note, T2Rs [148,149] and T1R3 [150] regulate intestinal tuft cells, which are apparently similar to the solitary chemosensory cells described below. Intestinal tuft cells express key components of taste signaling such as TRPM5 [131,151] and are key regulators of intestinal T helper 2 (Th2 or type 2) immunity in the gut by detecting parasites and other pathogens [152,153,154,155,156,157]. It is likely that taste receptors detect quorum-sensing molecules all over the body to signal information about bacterial population density to the host organism. Even the tongue type II taste cells themselves have recently been reported to regulate inflammatory cytokine production and may thus act as immune sensors [158]. Given the involvement of bitter and sweet taste receptors in detecting molecules of bacterial origin, it seems essential to study the potential impact of the genetic diversity of these receptors on susceptibility to specific bacterial infections, between individuals and even between species.

## 5. Interactions of T2Rs with *Pseudomonas aeruginosa* Acyl-Homoserine Lactone (AHL) and Quinolone Quorum-Sensing Molecules in Airway Ciliated Cells

The sinonasal cavity, which includes the nose and the four paranasal sinuses, is the entry point of inhaled air into the body. It has the important function of warming the inhaled/inspired air and removing unwanted particles to protect the integrity of the more delicate lower respiratory tract [167]. Thus, it acts as the first line of defense against any pathogen or debris present in the air [15,16,168].

The defense mechanisms of the sinonasal cavity are both physical and chemical. Inhaled particles and microbes can be removed through mucociliary clearance (MCC): they are trapped into the upper layer of mucus secreted by the mucosa, and subsequently eliminated into the aerodigestive tract by the beating of the epithelial cells’ cilia. MCC is the prime physical defense of the airway. Severe respiratory infections occur when MCC is impaired in primary ciliary dyskinesia through impairment of cilia function [169] or in cystic fibrosis due to altered fluid secretion and mucus rheology [170].

The innate immune system also secretes several antimicrobial peptides and molecules, such as defensins and nitric oxide (NO), that neutralize and prevent infections [168,171]. The impairment of these defense mechanisms can lead to chronic infectious states such as chronic rhinosinusitis (CRS). CRS is a multifactorial disease that includes the impairment of the MCC, resulting in mucus stasis and chronic inflammation and infection of the upper airway [168]. CRS significantly reduces patients’ quality of life and heavily weighs on the US health care system. CRS is often treated with frequent prescription of antibiotics (~20% of antibiotic prescriptions in adults), which can promote the selection of resistant bacterial strains [172,173,174,175]. Overall, it is estimated that the management of CRS accounts for over USD 8 billion in direct health care costs in the US [176], and these costs increase in cases of increased antibiotic therapy failures [177].

**Figure 3:**
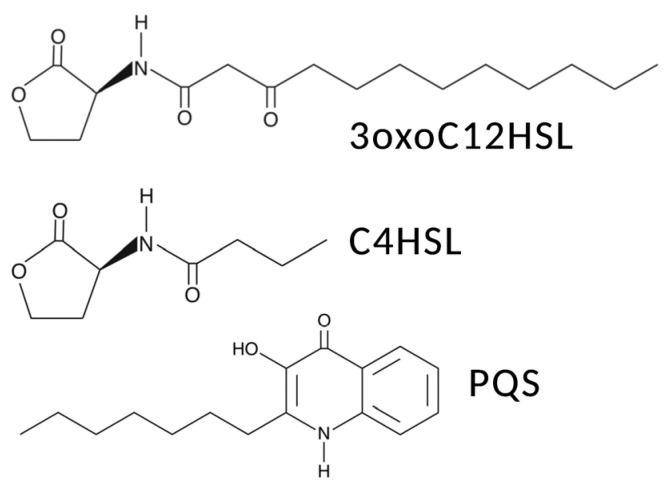
*Pseudomonas aeruginosa* quorum-sensing molecules shown to activate T2Rs, as described in the text. Shown are N-3-oxo-dodecanoyl-L-Homoserine lactone (3oxoC12HSL), N-butyryl-L-Homoserine lactone (C4HSL), and 2-heptyl-3-hydroxy-4(1H)-Quinolone, also known as *Pseudomonas* quinolone signal (PQS).

A novel approach in the treatment of CRS and other airway infections could be to explore bitter taste receptors as pharmacological targets by exploiting their role in innate immunity. T2R receptors are now known to be extensively expressed in the ciliated cells of the bronchial and sinonasal epithelia, specifically in the cilia themselves [16,161,178,179,180,181,182,183]. When activated by bitter agonists, they induce a Ca^2+^-dependent increase in ciliary beating frequency [184]. In the sinonasal cavity, this phenomenon could improve the elimination of pathogens and debris. Furthermore, whereas motile cilia (9 + 2 microtubule structure) were once thought to only have mechanical roles [185], the functional expression of bitter taste receptors in bronchial motile cilia suggests that they may also play a role in cell signaling similar to the primary cilia (9 + 0 microtubule structure, specialized in signal transduction and sensory functions). The T2R receptors that have been identified in ciliated sinonasal epithelial cells include T2R4, T2R14, T2R16, and T2R38 [16,160,161,178,179,180,181,182,183]. In airway cilia, T2R38 is specifically activated by N-acyl-homoserine lactones (AHLs), a family of molecules that are secreted and used as primary quorum-sensing signals by Gram-negative bacteria [19] (Figure 3).

**Figure 4:**
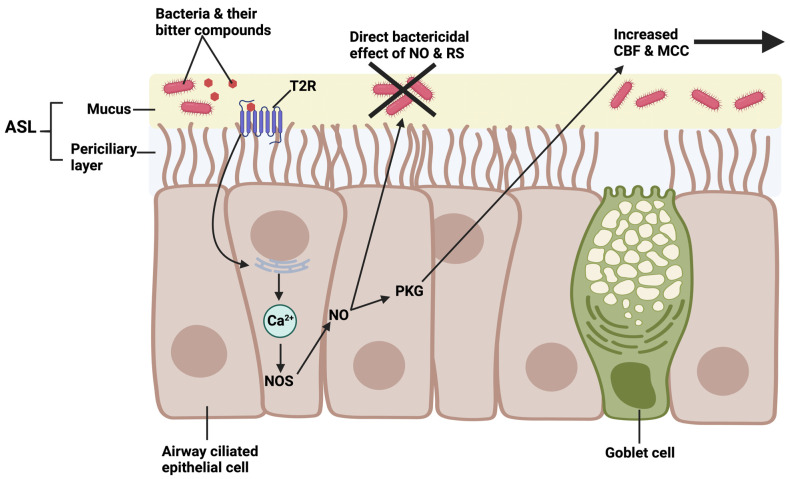
Role of cilia-localized T2Rs in the innate immune response of the sinonasal cavity. Inhaled pathogens and debris are trapped in the mucus lining the mucosa [15]. Gram-negative bacteria, such as *P. aeruginosa*, produce bitter agonists of T2Rs expressed in the airway, including acyl-homoserine lactones (AHLs) and quinolones [160,161]. Activation of T2Rs by these bitter agonists induces the release of Ca^2+^ stores from the endoplasmic reticulum. The elevation of intracellular Ca^2+^ leads to the stimulation of nitric oxide (NO) production by the enzyme nitric oxide synthase (NOS). The NO produced and its reactive species can diffuse into the airway surface liquid (ASL) and have a direct killing effect on bacteria, and also possibly on viruses and fungi, by destroying their cell wall. Through activation of protein kinase G (PKG) and subsequent phosphorylation of various ciliary proteins, the NO produced also increases cilia beating frequency, which improves mucociliary clearance [15]. Diagram created with BioRender.

In mice, AHLs activate nasal solitary chemosensory cells (SCC), hence their initial discovery as mouse nasal SCC agonists [186]. In humans, they appear to have little to no effect on nasal SCCs [134]. Instead, the stimulation of T2R38 by AHLs activates the Ca^2+^-dependent nitric oxide synthase (NOS) in ciliated cells, which leads to a strong increase in intracellular NO production, most likely mediated by the endothelial-named isoform eNOS [16,160,161,178,179,180,181,182,183]. The NO then increases the ciliary beating frequency and mucociliary clearance through activation of protein Kinase G (PKG) and the subsequent phosphorylation of specific cilia-localized proteins (Figure 4). Of note, this Ca^2+^ and NO signaling also involve PLCβ2 and TRPM5, as described above in the canonical type 2 taste cell signaling [16,160,161,178,179,180,181,182,183]. The NO produced may also have a broad-spectrum antimicrobial property, with demonstrated bactericidal activity against *P. aeruginosa* [16,160,161,178,179,180,181,182,183]. After production, the NO diffuses into the airway surface liquid (ASL) where it produces reactive nitrogen species (RNS). Both NO and its RNS can kill bacteria by damaging their membranes, cell walls, and DNA, as well as by inactivating a wide range of other proteins. NO may also have fungicidal and antiviral properties by damaging various viral envelope proteins and fungal cell walls [187,188,189,190].

Because of the existence of *TAS2R38* polymorphisms, it is consistent to hypothesize that *TAS2R38* genetics may influence the magnitude of the T2R38-mediated immune response, depending on patient genotypes. In a first step to test this hypothesis, sinonasal epithelial cells from PAV (super-tasters) and AVI homozygous patients (non-tasters), as well as from PAV/AVI (intermediate tasters) patients, were cultured in vitro at the air-liquid interface (ALI), and their level of Ca^2+^ signaling and NO production were investigated [160]. We demonstrated a significant correlation between these two parameters and the specific genotypes of each patient from whom the cells were collected [160]. In addition, PTC- and AHLs-stimulated super-taster (PAV/PAV) cells exhibited significantly higher levels of Ca^2+^ signaling and NO production, as well as enhanced MCC and bactericidal effect, compared with non-taster (AVI/AVI) and intermediate taster (PAV/AVI) cells [169]. Overall, these in vitro results indicated that *TASR38* taste-altering polymorphisms also modify the sinonasal epithelial cells’ responses to Gram-negative bacteria su8ch as *P. aeruginosa*. They were subsequently reinforced by clinical studies that showed super-tasters were less likely to develop Gram-negative sinonasal infections than non-tasters and intermediate tasters [160]. Regarding severity factors and outcomes, AVI homozygous patients tend, overall, to develop severe forms of CRS requiring functional endoscopic sinus surgery (FESS) more frequently [191,192], and bacteria isolated from their sinonasal cavities have a higher frequency of developing biofilms in vitro [193]. AVI/AVI patients who develop CRS without nasal polyps have worse clinical outcomes after FESS, whereas PAV/PAV patients have better outcomes if they receive FESS [178].

Following the results described above, a growing number of studies have continued to examine the relationship between *TASR38* polymorphisms and CRS [194,195,196,197,198,199,200,201]. One study conducted in an Italian population did not find a correlation between CRS and *TAS2R38* polymorphisms [194]. These conflicting results could be due to differences in study populations, as the patients included in this study had more refractory clinical pictures of CRS with exacerbated Th2 inflammation components. In contrast, a recent study found a correlation between severity of CRS and PAV and AVI variants in a Polish population [195], an Australian study demonstrated a correlation between non-taster genotype (AVI/AVI) and presence of culturable bacteria colonizing the sinuses [198], and another Italian study found that non-functional T2R38 correlated with in vivo infections by Gram-negative bacteria and with the presence of biofilms in patients diagnosed with CRS with nasal polyps [199]. Genomic-wide association studies (GWAS) seem to also support a relationship between *TAS2R38* genotype and CRS. A Canadian GWAS found that patients with CRS more frequently had SNPs in the bitter receptor genes *TAS2R38* and *TASR13* than their non-diseased counterparts [196], and a US study demonstrated a correlation between CRS and SNPs in *TAS2R38* and *TAS2R19* genes [197].

**Figure 5:**
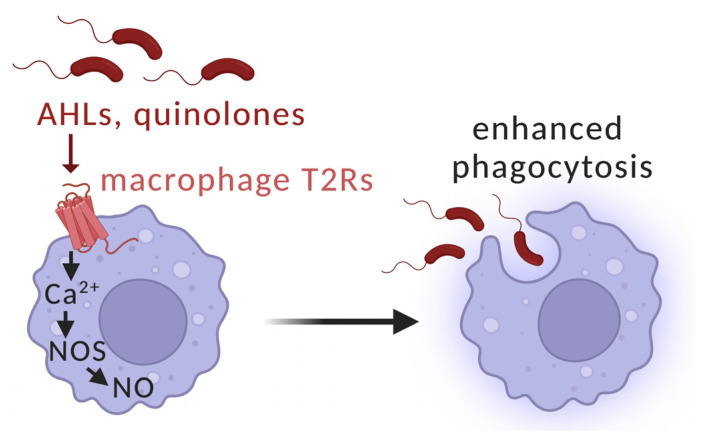
Macrophage T2R detection of bacterial AHLs or quinolones results in Ca^2+^-dependent NO production and enhancement of phagocytosis through protein kinase G [181,183,209]. Diagram created with BioRender.

Recent studies demonstrate that T2Rs are true immune recognition receptors, much like Toll-like receptors (TLRs). Gram-negative AHLs have been shown to be able to activate other T2Rs than T2R38, including isoforms T2R10 and T2R14 when expressed in HEK93 cells [202]. In addition to their expression in sinonasal epithelial cells, T2Rs were also identified in immune cells where they may contribute to the detection of quorum sensing molecules [203,204,205,206,207,208]. We demonstrated that unprimed macrophages (M0) can be activated by AHLs or quinolones through T2R receptors. This activation led to enhanced bacteria phagocytosis through the same T2R-eNOS pathway used by airway ciliated cells and previously described in this paper [181,183,209] (Figure 5).

Other quorum-sensing molecules that may activate T2R-mediated immune response in airway ciliated cells include two quinolones also produced by *P. aeruginosa* and currently unidentified molecules present in media conditioned with *Bacillus cereus* [84]. In the sinonasal cavity, T2R receptors on ciliated cells may therefore act as proper immune receptors and participate in antimicrobial surveillance by detecting specific bacterial bitter compounds produced at different growing stages (e.g., planktonic vs. microcolony vs. biofilm). This monitoring may allow epithelial cells to differentiate commensal carriage from pathogenic bacterial growth and help prevent/control the development of infections by eliminating invasive species as they begin to produce detectible quorum-sensing molecules [85,86].

Cystic fibrosis (CF) is a rare and fatal disease caused by a defective mutated cystic fibrosis transmembrane regulator (CFTR) protein, an anion channel that regulates fluid secretion in glandular organs such as the lungs, pancreas, and reproductive organs [170]. In CF patients, *P. aeruginosa* is a major cause of morbidity and mortality and promotes rapid decline in respiratory function [210]. The involvement of *P. aeruginosa* in the natural history of CF and its ability to produce T2R38 ligands led scientists to investigate the relationship between susceptibility to this bacterium and *TASR38* genotype in CF patients. Studies on that subject have yielded conflicting results. The initial study, which showed correlation between *TAS2R38* genotype and some CRS-related symptoms in CF patients [211], was not corroborated by its first follow-up study, which found no correlation between *TASR38* and infection by *P. aeruginosa* in CF patients [212]. Another study published more recently found that the frequency of the *TAS2R38* PAV allele was significantly lower in CF patients who had CRS with nasal polyps and required FESS [213]. It also subsequently found that the frequency of the PAV allele was lower in CF patients whose lungs were chronically colonized by *P. aeruginosa*, especially when this colonization occurred before the age of 14 years [213].

Overall, these previous studies suggest that the PAV allele may confer subtle protection against CRS and possibly infection by *P. aeruginosa* in CF patients. The low impact of *TAS2R38* genotypes in CF may be rooted in the altered NO responses that have been described in CF patients [183]. In this population, eNOS signaling in endothelial cells, as well as T2R-mediated NO production in response to AHLs in nasal primary nasal cells are impaired and/or reduced [183,214]. The improvement of CFTR function in CF patients using a corrector/potentiator combination led to restoration of NO production in primary nasal cells [183]. Altered CFTR function may therefore be responsible for a decreased innate immune response in CF patients, independent of their *TASR38* genotype, contributing to their high susceptibility to *P. aeruginosa* and reducing the influence of *TAS2R38* on CF-related infections. With current CF treatments using CFTR modulators to restore the ion channel function in patients [210,215], we may begin to observe a greater impact of *TASR38* genetics on the susceptibility of CF patients to *P. aeruginosa* infections.

T2R-mediated NO production is also reduced during co-stimulation of NPY, which activates inhibitory protein kinase C phosphorylation of eNOS through NPY2R receptors in ciliated epithelial cells [180]. As levels and/or density of NPYergic neurons may be increased in some sinonasal diseases, this may be a mechanism by which beneficial T2R innate immune responses are reduced during epithelial inflammation disease. Notably, the T2R-dependent NO production requires functional heat shock protein 90 (HPS90), which can bind to and interact with eNOS [181]. HSP90 inhibitors have been proposed as therapeutics for airway remodeling and goblet cell metaplasia observed during Th2 inflammation in airway diseases such as asthma [216]. A potential off-target effect of such therapies might be unwanted reduction of T2R/eNOS-mediated NO production from ciliated cells [181].

## 6. Interactions of Bitter Taste Receptors in Gingival Epithelial Cells with *Streptococcus mutans* Competence Stimulating Peptides

Gingival epithelial cells express T2R14, which was recently identified to be involved in detecting competence stimulating peptides (CSPs) from Gram-positive *Streptococcus mutans* [30], a common oral bacteria that causes dental carries [217]. *S. mutans* CSP-1 activated intracellular Ca^2+^ signaling and secretion of IL-8 (CXCL-8), TNF-α, and IL-6 in gingival cells. This was blocked by a T2R14 antagonist (6-methoxyflavone) or CRISPR/Cas9 knockout of T2R14 [30]. Interestingly, another study by the same group showed that knockdown of T2R14 reduced internalization of *S. aureus* but not *S. mutans* in their gingival epithelial cell model [31]. When gingival cells were primed with *S. mutans* CSP-1, they observed inhibition of growth for *S. aureus*, but not for *S. mutans* [31]. This was explained by a T2R14-dependent activation of secretion of potent antimicrobial β-defensin-2 (hBD-2) with *S. aureus* but not *S. mutans* [31]. Instead of hBD-2 secretion, *S. mutans* induced T2R14 dependent IL-8 secretion [31].

Furthermore, they showed that T2R14 knockout reorganizes the cytoskeleton in gingival cells, potentially explaining the inhibition of *S. aureus* internalization [31]. While these results show exciting differential interactions between bacteria species and T2R14, their full implications are not yet clear. The CSP-1 stimulated GECs attracted differentiated HL-60 cells, a leukemia line used to model monocytes and macrophages, in a T2R14-dependent manner. However, the full context of the differential interactions requires animal model studies where the full crosstalk between innate and adaptive immunity is preserved. Because *S. mutans* and *S. pneumoniae* share similar CSP quorum-sensing mechanisms [29], there may be important implications for CSP-T2R14 interactions in upper and lower respiratory tract *S. pneumoniae* infections [218,219].

Nonetheless, these data suggest that T2R14 in the oral cavity might be a target for stimulation of innate immune responses in patients with gingivitis or certain types of gingival infections. This may extend to other bitter receptors. A meta-analysis of GWAS data suggested that *TAS2R43* and *TAS2R14* gene expression levels are associated with early childhood dental caries [220]. Another study showed that variants in *TAS2R3*, *TAS2R4*, *TAS2R5*, and *TAS2R60* are also associated with severe early childhood caries [221], finding that variant taste genes were also correlated with relative abundances of bacteria and fungi [221]. *TAS2R38* PAV and AVI polymorphisms have been liked to oral microbial composition by 16S rRNA sequencing [222]. T2R16 activation in oral gingival fibroblasts has been suggested to reduce pro-inflammatory cytokine production downstream of lipopolysaccharide and NF-κB signaling [223].

## 7. Interactions of Sweet Taste Receptors with Bacterial D-Amino Acids in Airway Solitary Chemosensory Cells

Solitary chemosensory cells (SCCs) are a type of specialized, elongated chemosensory cell that makes up about 1–10% of the cells in the sinonasal cavity [74,132,133,134,186,224,225,226,227,228,229,230]. They are also called tuft cells” or “brush cells” because they sometimes have an apical tuft of microvilli [231,232]. SCCs were most probably identified for the first time in rats [233,234,235] and later in humans’ lower airways by electron microscopy [236,237]. Very little is currently known about their functions and signaling pathways, although they appear similar to those of intestinal tuft cells [152]. Little is known either about their role in diseases of the respiratory tract. Further investigation of human airway epithelia cells using transcriptomic approaches could help us better understand the physiology and even potential heterogeneity of human SCCs [231,238].

**Figure 6:**
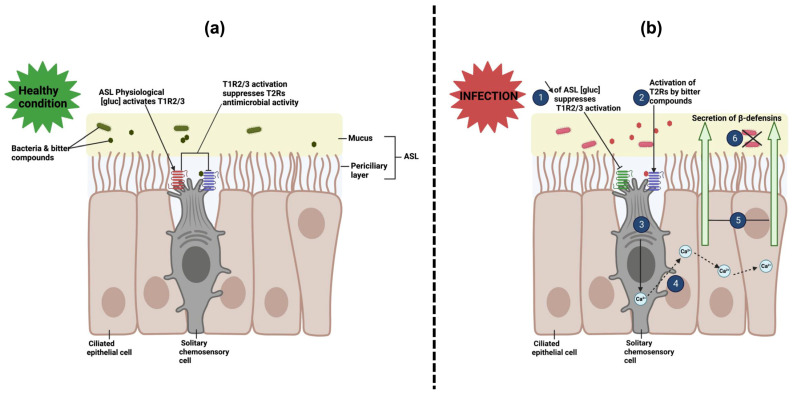
Role of sweet (T1Rs) and bitter (T2Rs) taste GPCRs expressed in solitary chemosensory cells (SCCs) in human sinonasal innate immune response. (**a**) Under healthy conditions, the physiological concentration of glucose in the airway surface liquid (ASL) activates T1R2/3, resulting in the repression of T2R-mediated antimicrobial activity in the same SCC [74,134]. (**b**) During infection, a decrease of glucose levels outside the activation range of T1R2/3 (0.5–5 mM) leads to the inactivation of T1R2/3 and subsequent activation of T2R. This results in a Ca^2+^-dependent release of β-defensins 1 and 2, antimicrobial peptides that kill Gram-positive and Gram-negative bacteria, from surrounding epithelial cells [74,134]. Diagram created with BioRender.

Upper airway SCCs have been shown to co-express both bitter (T2R) and sweet (T1R2/3) taste receptors in a single cell, in contrast to type II taste bud cells that only express one type of taste receptor each [104,239]. Activation of T2Rs in SCCs can yield different results, depending on the species. In mice, for instance, interaction of T2Rs with bitter agonists can generate neurogenic inflammation [230] and reflex retention of breath through the activation of trigeminal afferent nerve fibers [133]. In humans, sinonasal SCCs have been localized in the septum, uncinate process, middle and lower turbinates, and nasal polyps [132,240]. Activation of SCC T2R receptors appears to be involved in the innate immune response against bacteria; it generates immediate secretion by surrounding epithelial cells of antimicrobial peptides that kill both Gram-positive and Gram-negative bacteria (β-defensins type 1 and 2, specifically) [74,134] (Figure 6). Of note, SCCs and ciliated epithelial cells express different isoforms of T2Rs (T2R4, 14, 16, and 38 for ciliated cells and T2R10, 46, 47, for SCCs). Bacterial agonists that can activate human SCCs T2R have yet to be identified.

Sweet taste receptors in SCCscan be activated by several types of molecules, including artificial sweeteners and glucose at defined concentrations (0.5–5 mM). Interestingly, this activation counteracts T2R signaling in the same cell and reduces the antimicrobial response by decreasing the release of β-defensins [74,134]. Scientists have hypothesized that the attenuating effect of glucose may act as a “safeguard” against the elimination of commensal bacteria that also produce bitter compounds under otherwise healthy physiological conditions. Indeed, under physiological conditions, the ASL glucose level is within the concentration range that activates T1R2/3 SCCs, effectively attenuating T2R activation. Under pathological increase in bacterial density, the increased production of bacterial bitter agonists and concomitant decrease in glucose concentration in the ASL (consumed for bacterial metabolism) [241] inactivates T1R2/3 in SCCs, allowing for T2R activation, secretion of antimicrobial peptides, and clearance of the infection.

While the mechanism described above may be beneficial in healthy patients, it may negatively impact airway disease in CRS patients with comorbidities that can also alter the physiological concentration of glucose in the ASL. Healthy ASL glucose concentration, which is about 0.5 mM, arises from tonic leakage of epithelial glucose from the serous fluid and is 10 times lower than the resting serum glucose concentration. CRS patients with an impaired epithelial barrier due to inflammation [134,242] and diabetics with hyperglycemia [241] have ASL glucose concentrations above the physiological value (≥3–4 fold). In such cases, topical treatment with T1R2/3 antagonists, such as lactisole [83], could reduce the T1R response related to ASL elevated glucose levels and restore acceptable T2R immune responses in some patients.

A key distinguishing mechanistic factor between the nasal SCC T2R responses and the nasal ciliated cell T2R responses is the requirement for the taste- and tuft-cell specific Gα-gustducin, the transducin-like Gα involved in taste signaling [38,243,244]. Similar to intestinal tuft cells [245], SCC T2R responses appear to require gustducin [74,134], whereas the T2R NO responses from ciliated cells do not [160,178]. While T2Rs couple to Gα-gustducin in taste and taste-like cells, T2Rs have also been shown to couple to Gαi subunits in airway smooth muscle [246]. As many GPCRs exhibit a degree of G protein promiscuity [247], it is likely that T2R signal transduction can make use of different G proteins based on the relative affinities of the available G proteins expressed in an individual cell type. This differential requirement for Gα-gustducin can allow experimental determination of tuft-cell-dependent and tuft-cell-independent responses.

Besides glucose and artificial sweeteners, some bacterial D-amino acids have also been identified as T1R2/3 agonists in human sinonasal SCCs. Gram-positive and -negative bacteria, such as *Bacillus subtilis* and *Vibrio Cholerae*, typically produce a wide variety of D-amino acids for the synthesis of their cell wall peptidoglycans, and these molecules may also serve as quorum-sensing signals [248]. While the literature is replete with studies on the potential role of D-amino acids as quorum-sensing signals, these studies are not always consistent and are difficult to compare with one another because they are not standardized (e.g., different bacterial strains, biofilm assays, D-amino acids investigated). Some researchers have proposed that D-amino acids may be involved in biofilm disruption mechanisms in bacteria, including inhibition of formation in species such as *P. aeruginosa* [249,250], *S. aureus* [251,252], and *Staphylococcus epidermidis* [253], and disassembly, dispersal, or detachment of preformed biofilms in *B. subtilis* [254], *P. aeruginosa* [255], *S. epidermidis* [253], *S. aureus,* or in mixed-species systems [256,257]. In addition, *P. aeruginosa* and *S. aureus* sensitivity to rifampicin may be increased by D-amino-acids [255].

Among studies that temper or even contradict those described above, one suggests that D-amino acids indirectly inhibit *B. subtilis* biofilm formation by interfering with protein synthesis instead of disrupting the pre-formed biofilm [258]. Another study reported that D-serine was able to inhibit biofilm formation in *Mycobacterium tuberculosis*, but found no such influence of D-amino acids at 1mM in *S. aureus*, *B. subtilis*, or *S. epidermidis* [259]. This study also found no reduction in biomass of pre-formed biofilms with D-serine, D-alanine, D-valine, D-phenylalanine, nor D-threonine. It was suggested that amino-acid dosages may influence biofilm mass in different species [260], and that mixtures of D-amino acids [261] or combinations with antibiotics [262] may potentiate their anti-biofilm effects. Considering these conflicting results, further and better standardized studies are needed to unveil the role of D-amino acids in bacteria physiology.

Bacteria typically produce D-amino acid at high concentration ranges (µM-to-low mM) [248,254,263,264,265,266,267] where they may taste sweet through activation of T1R2/3 on the tongue [72,268]. We showed that *S. aureus* and coagulase-negative *Staphylococcus* cultures isolated from the sinonasal cavities of CRS patients produced D-Leucine and D-Phenylalanine in concentrations sufficient to activate T1R2/3 in SCCs. This activation resulted in a decrease in T2R-mediated response in SCCs and in the overall immune response of the airway epithelium in vitro. One theory is that this repression of immune response may be another “safeguard” mechanism to prevent clearance of commensal *Staphylococcus* species in vivo. This theory is supported by the apparently crucial role of bacterial D-amino acids in communication between bacteria and host cells [269]. However, this mechanism may also allow pathogenic *Staphylococcus* to escape immune detection in the respiratory tract. For the moment, neither of these two hypotheses has been proven to be the most probable.

Bacterial D-amino acids may have a broader implication in the regulation of human immunity. In a recent preprint, we reported that D-amino acids’ interaction with T1R3 in ciliated cells may reduce ASL glucose, potentially resulting in impeded bacterial growth [163]. Another study hypothesized that D-amino acids may be involved in the regulation of B cell and macrophage function in the intestine [270]. More research is therefore needed to better understand the role of D-amino acids in human immunity, particularly through their interaction with T1R receptors. The importance of *TAS1R* genetics in airway disease, including how it alters D-amino acid activation of the T1R SCCs, innate immunity, and CRS outcomes, should therefore also be investigated.

Finally, SCCs appear to be major effectors of CRS inflammation by producing IL-25, an early signal for type 2 airway inflammatory responses [168,271]. Their density was shown to increase in tissues from patients with allergic fungal rhinosinusitis, and SCC expansion and differentiation appeared to be stimulated after exposure to fungal extracts in vitro [272]. In mice, SCCs are mainly localized in the nose [273] and trachea [227,274], and have also been described in the distal lungs after severe influenza infections. It is possible that the proliferation and differentiation of SCCs under inflammatory stress involves the activation of taste receptors by fungal or even viral quorum-sensing signals [275,276].

## 8. Conclusions and Remaining Questions

The idea of immune cell receptors surveilling bacteria is not new. The ubiquitous Toll-like receptors (TLRs) and other pattern recognition receptors detect bacterial-specific components such as lipopolysaccharide (TLR4) or flagellin (TLR5) [171]. In light of this, it is not surprising that chemosensory receptors including taste and olfactory receptors also participate in the detection of bacteria. One important aspect of T2R bitter receptors that makes them therapeutically attractive is the wide range of clinically-used compounds with existing safety data that are known to be bitter [135,137,277]. Such drugs may be repurposed to activate these receptors to stimulate defense antipathogen immune responses [278,279]. Moreover, many plant compounds are also bitter [179,280,281,282,283,284], and activation of T2Rs may explain particular beneficial effects with some homeopathic plant-based therapies. The drive to develop artificial non-metabolizable sweeteners has also led to many compounds known to activate the sweet taste receptor that might also be leveraged therapeutically [38,67].

One area that is lagging in taste receptor research is specific antagonists. While a recent study has developed promising antagonists for T2R14 [285], most known T2R inhibitors are poorly characterized against other isoforms, are usually not isoform specific, and have low affinities raising concerns about off-target effects [284,286,287,288,289]. Development of more specific, higher affinity agonists and antagonists will allow better dissection of the molecular details of the contributions of individual T2R isoforms. While an allosteric inhibitor of human T1R3, lactisole [83], is frequently used in T1R studies, this compound has off-target effects on cAMP and should thus be used with caution [290,291].

It is likely that more currently unknown bacterial compounds activate T2Rs. An important question is whether only potentially pathogenic bacteria activate T2Rs or if generally non-infectious related bacteria (e.g., *S. aureus* vs. *S. epidermidis*) also interact with host T2Rs. It may be that T2Rs contribute to detection of “good” vs. “bad” bacteria. An alternative is that T2Rs could be activated by any bacteria that reach a density high enough to secrete enough bitter metabolites that activate T2Rs. While many diverse compounds can activate these receptors, T2R isoforms expressed in various tissues might create some degree of specificity for certain molecules. All of these questions need to be examined in the future to better understand how T2Rs fit into host–pathogen interactions. Another class of metabolites that should be investigated include various “quorum quenching” molecules [22,24] which are being developed to inhibit bacterial quorum sensing in the context of infections and biofilm formation.

An important remaining question is whether fungi or viruses also activate bitter taste receptors. Like bacteria, fungi produce a host of metabolites, many of which are likely to be bitter. Whether they reach sufficiently high concentrations to activate T2Rs or T1Rs remains to be determined. Given that taste-receptor-expressing tuft cell/SCC abundance in the upper airway is increased with Th2/type 2 immunity and fungal exposure [271,272,292], it appears likely that taste receptors may play some role in fungal infections. Fungal proteases have long been known to activate GPCRs such as protease activated receptor 2 (PAR-2 [293,294,295]), so other fungal–GPCR interactions may occur, possibly through taste receptors.

Do T2Rs play any role in viral infection? SCC/tuft cell abundance has been shown to increase in the distal lung after viral infection [296]. However, given that viruses do not themselves produce the same repertoire of metabolites compared with bacteria and fungi, it is harder to speculate how viruses could activate taste receptors. However, because peptides can be T2R agonists [30,297] or blockers [286,298,299], it is in theory possible that viral proteins can activate one or more taste receptors. It may also be that taste receptors detect metabolites released by cells during viral replication or budding or during viral-induced apoptosis. Future studies are needed to determine if and how there are viral-taste receptor interactions.

The localization of T2R14 and other T2Rs in oral epithelial cells may also have implications for host–microbe interactions in oral cancer. We showed that bitter receptors, including T2R14, are expressed in oral keratinocyte-derived cancer cells, where they activate apoptosis [300]. Moreover, analysis of The Cancer Genome Atlas showed increased expression of *TAS2R* genes associated with improved overall survival in head and neck squamous cell carcinoma [300]. *TAS2R* and *TAS1R* genes are also associated with survival in many solid tumor types [301], suggesting that these genes are potential biomarkers to predict cancer patient outcomes or treatment responses. Importantly, it suggests that T2Rs may mediate tumor–microbiome crosstalk. Such a link may occur between T2R and CSPs in oral cancer. Because the same T2R-driven apoptotic pathways are also found in lung cancer cell lines [182], such interactions may occur through *Pseudomonas* AHLs or quinolone with T2R4, T2R14, or T2R38. Several other studies have shown functional expression of T2Rs in cancer and/or genetic associations of TAS2R or TAS1R genes with cancer [302,303]. Further studies of the roles of T2Rs in detecting quorum-sensing molecules are needed to help clarify how T2Rs influence tumor–microbiome crosstalk [304,305].

## Figures and Tables

**Table 1:** Bacterial strains shown to produce human taste receptor-activating compounds.

Bacteria Species	Strain(s)	Molecule Detected by Taste Receptors	Effects
*Pseudomonas* *aeruginosa*	PAO1, Sad36 [159]	Acyl-homoserine lactones (AHLs)	T2R activation, increased nasal cell nitric oxide production, ciliary beating, bacterial killing [160]
	PAO1	*Pseudomonas* quinolone signal (PQS)	T2R activation, increased nasal cell nitric oxide production, ciliary beating [161]
*Staphylococcus aureus*	M2 [162]	D-amino acids	Activation of T1R2/3, suppression of solitary chemosensory cells [74]
	M2, clinical isolates	D-amino acids	Activation of T1R3, increased airway glucose transporter expression [163]
	M2	unknown	T2R activation, increased nasal cell nitric oxide production [163,164]
*Streptococcus* *mutans*	UA159	competence stimulating peptides	T2R activation, cytoskeletal remodeling, increased bacterial internalization in gingival keratinocytes [31]
*Bacillus cereus*	ATCC 14579	unknown	T2R activation, increased nasal cell nitric oxide production
*Bacillus* *amyloliquefaciens*	amy-1 [165]	exopolysaccharides	T2R activation, glucagon-like peptide 1 secretion [166]

## Data Availability

No new data were created or analyzed in this study. Data sharing is not applicable to this article.
